# Analysis of adequacy levels for human resources improvement within primary health care framework in Africa

**DOI:** 10.1186/1478-4505-3-8

**Published:** 2005-12-02

**Authors:** Florence Parent, Audrey Fromageot, Yves Coppieters, Colette Lejeune, Dominique Lemenu, Michèle Garant, Danielle Piette, Alain Levêque, Jean-Marie De Ketele

**Affiliations:** 1 Department of Epidemiology and Health Promotion, School of Public Health, Université Libre de Bruxelles (ULB), Brussels, Belgium; 2 UMR PRODIG, Paris, France; 3 Institut Supérieur d'Enseignement Infirmier (ISEI), Brussels, Belgium; 4 Centre de Pédagogie Universitaire, Université Catholique de Mons (FUCAM), Belgium; 5 Education Department, Université Catholique de Louvain (UCL), Belgium

## Abstract

Human resources in health care system in sub-Saharan Africa are generally picturing a lack of adequacy between expected skills from the professionals and health care needs expressed by the populations. It is, however, possible to analyse these various lacks of adequacy related to human resource management and their determinants to enhance the effectiveness of the health care system. From two projects focused on nurse professionals within the health care system in Central Africa, we present an analytic grid for adequacy levels looking into the following aspects:

- adequacy between skills-based profiles for health system professionals, quality of care and service delivery (health care system /medical standards), needs and expectations from the populations,

- adequacy between allocation of health system professionals, quality of care and services delivered (health care system /medical standards), needs and expectations from the populations,

- adequacy between human resource management within health care system and medical standards,

- adequacy between human resource management within education/teaching/training and needs from health care system and education sectors,

- adequacy between basic and on-going education and realities of tasks expected and implemented by different categories of professionals within the health care system body,

- adequacy between intentions for initial and on-going trainings and teaching programs in health sciences for trainers (teachers/supervisors/health care system professionals/ directors (teaching managers) of schools...).

This tool is necessary for decision-makers as well as for health care system professionals who share common objectives for changes at each level of intervention within the health system. Setting this adequacy implies interdisciplinary and participative approaches for concerned actors in order to provide an overall vision of a more broaden system than health district, small island with self-rationality, and in which they operate.

## Introduction

The organization of health systems in sub-Saharan Africa is, more than elsewhere, born in close connection with the establishment of political and territorial structures, initially within the colonial framework, then within the building of independent States [1,2]. Since 1980s, the economic and financial crisis of several States is marked by their disinvestments in the development and planning of programs associated with the promotion decentralization models [3]. Inspired by WHO taken over by the World Bank in 1990s, the systems of care are organized at the base, within and around Health districts liased with "minimum and complementary packages for care" to provide better answers to populations' requests [4]. Within this framework, many questions persist concerning improvement of medical systems, focused mainly if not exclusively, on financial and organizational techniques. In view of these persistent inefficiencies, priorities for medical actions in sub-Saharan Africa usually reinforce mechanistic approaches where an overall vision of the whole situation is eluded in favour of an approach covering separately different operational sectors in the fields of planning, training, implementation and evaluation.

The management of human resources in health more often participates to the sustainability of an "inhospitable medicine" in Africa [5]. It is however a recent stake in the rich as well as in the poor countries [6]. Within health systems, it represents health care implementation. It questions practices, their findings and efficiencies in the heart of interactions between various actors concerned: professionals and populations. Since 1990s, it remains an object of increasing concern of works and thoughts on improvement of the effectiveness of health care structures [7]. The case studies are becoming more sensitive on human resources and their management in the health systems, especially in Africa [8,9] where, more than elsewhere, the quality of care seems to be lacking, in the actions as well as in the perceptions from the populations who have poorly recourse to it [10,11].

In sub-Saharan Africa, actors and observers agree in recognizing the discordances and inefficiency of health care practices while intensifying many national programs for building human resources' capacity. These projects, however, rarely adopt a global approach to needs and roles for health care professionals relocated in all sectors of interventions such as medical, educational or planning. In this article, we underline the need to consider health care professionals in their interactions with all the actors in the health environment in one area at a given time. These stakes are translated early in the 21st Century by a redefinition of the organization of health districts around their human resources. This public health objective is at the core basis of projects and action plans aiming particularly at a better adequacy between on the one hand the offer of on-going training (specific and on-the-job training) or initial (initial training curriculum), and, on the other hand, expectations from health care professionals as well as the needs for the populations.

The approach on organization of health care systems in Africa by its human resources management supposes the assertion of new frameworks of analysis and extended action to political, sociological, educational and motivational dimensions. Discussion on human resources management appears then in all the complexity of its multi-factorial dimensions using not only the health objects for which these resources exist, but also areas of training, planning and sociological determinants related to actors' behaviours. If approaches in more systemic terms of health districts and their actors have already been initiated [12], the consideration of links between these various dimensions still remains insufficient.

The article defines in an original way these multi-factorial and multi-level links corresponding to levels of adequacies or organizational inadequacies, determinants from the coherence and effectiveness of health care systems in sub-Saharan Africa. It recommends a new framework of analysis and understanding of these forms of (in)adequacy on human resources' management in relation to expected competences from professionals and the needs for the populations. Planning this conceptual framework based on field works in Democratic Republic of Congo (DRC) and in Rwanda plays a role in improving medical systems, in general, and structuring primary health care in particular.

## Methods

### Development of the conceptual framework

Thinking on human resources in the health sector in sub-Saharan Africa leads to develop adapted tools. The first step consists in context-oriented human resources and their management within the entire health system and its actors, internally as well as externally. As recent works remind it, for the comprehension of the social world, to extract any element from its context is eminently dangerous. In fact we face a health system in which it becomes essential to replace all interrogations about the place and role of the professionals [11]. The human resource management should be relocated within the entire aspects of the organizational modalities and improvement of medical systems (States' policies to staffs and populations of therapeutic practices and their initial trainings) to provide answers to recurring interrogations which raise concern and difficulties to obtain quantitative and qualitative adequacies for health care professionals with the needs of objective and perceived care for the populations. This questioning is not added to many explanations on dysfunctions of the African medical systems but opens way to the formulation of objectives for changes aiming at a better effectiveness in the health care system.

The search for a greater effectiveness regarding nursing practices and their recourse opens to the second step. This one is the opposite to break with actions burst in sectors of technical performances and scattered in distinct skills. In the approach to improve medical systems, there is not question any more to dissociate spatio-temporal dimensions in liaison with sectors of operation separating, for example, the local and immediate levels of operative functions for health care structures, on the one hand, and levels including organization and decision of the health care systems, on the other hand. These approaches by sectorial activities produce changes, which can only intervene within the medical system, without modifying its structure and its functioning, nor the links and their effects between its elements.

These two steps are found within a systemic approach of human resources in the health care sector. The systemic vision, supported by a research-action inter-sectorial approach, puts in perspective human resources in its interactions with all components of the medical systems. It opens a way towards a representation of changes, which suppose the overall progress of the system, to which they apply. The aim is the development of a new model of knowledge from the two human resources capacity building projects in central Africa concerning the field of health:

The first project, initiated three years ago in the Democratic Republic of Congo (DRC), recommends to support initial teaching in health sciences in the secondary level of education at the national level. In a first stage, the project develops coherence, relevance and understanding for a significant number of actors and stakeholders of strategic orientations, founders of the reform of the nurse sciences program required by the Department of Health Sciences Education within the Ministry of Health. In 2005, this reform is on the way. The whole process enables autonomy of teachers, as well as of learners, managers, department staff and supervisors.... It is by a methodological work calling upon concepts such as active pedagogies, skilled-based approach [13,14], organizational learning, thinking and self-assessment, built by partnership and interrelationships between all the actors together, giving greater importance to improving health care quality and their perceptions within the framework of Structures for primary health care.

The second project in progress is a national support to schools of nurse sciences in Rwanda. The steps and methods are similar to those launched in DRC. The interest carried to human resources passes by a second reading of the training package related to health sciences (professional, higher and academic levels). The search for a better adequacy between trainings and health professional expectations as well as those of the public regarding care, underlines the necessity to train nurses in technical secondary level on the skill-based approach. The project is also involved in an in-depth work with the various local and national actors: teachers, internship supervisors, directors of educational establishments, learners, and Ministries. It articulates, indeed, several organizational and institutional levels: local learning environments complying with medical standards, human resource requirements planning and training schemes.

When projects for general thinking are located and specific to a category of professionals (nurses), actors and fields are relocated in the entire medical environment including the populations, social and political supervisors. The stake is not just the detailed observation of actors and their relations with the health care systems, but to go *in fine *beyond traditional explanatory models of health care (dis)functioning in Africa focused on districts. The different sites contribute by developing an analysis framework on more complex realities than simple setting in linear equation between, on the one hand, the medical standards planned by national institutions, and, on the other hand, the assessment of local requirements in human resources without integration neither for their training modalities nor for the expectations expressed by the populations.

A grid of analysis is suggested where human resource management, including for nurses of primary health care structures, falls under the overall medical system and the diversity of its political actors, health care professionals or not. These components are considered within their dynamic interactions, as much undergone as built. It makes it possible to avoid separating artificially human resource management, perspectives for planning, training education, and evaluation. Persistent dichotomies between spheres of health and education are checked through penalizing field-based discordances between professionals' skill profiles and their needs while meeting populations-expressed expectations. The perspectives for efficient changes of a health system assumes improving different adequacy levels which are to avoid reduced searching " for oasis of rationality" [12], limited to dimensions of each health district, and to the implementation of sectorial projects launched together in space and time.

## Results

### Presentation of the conceptual framework

The diagram constitutes a grid of analysis of levels of (in)adequacies in human resources management in health system with different components and organization methods for health care systems taking part in its (dis)functioning. The structure obtained is prompted by the articulation between levels and system organization sectors: States producing heath care system standards, with planning programs and health care system management and its professionals, and local actors and their training for care practice.

The elements are illustrated by the six "boxes" which define the major levels of adequacy between the management of the health care professionals and ways of improvements, in terms of quality of care and health system effectiveness. The arrows show links, i.e. the interdependences between these forms and levels of adequacy. The structure is provided by the overall composition of the diagram.

Elements, links and structure account for a "construction of health":

- Field of action of national policies inspired from international standards;

- Societies' stake and their Community participation;

- Object of local implementation of care.

These various levels of organization of health care system appear through three "sectors" shown in the diagram: from macro level developing qualitative and quantitative health care standards (the "Health sector" of the national and international policies concerned), to the meso, spaces and actors of the medical training registered in projects of companies like the "sectors of planning and of the human stock management" and, to the microphone of the daily practices of health whose forms of application depend directly on the "sector basic training" of the professionals.

These sectors show the importance of levels for observation, stakes and human resources analysis, as thoughts of social sciences in the field are concerned of articulation modalities between macro and microanalyses levels of standards and health care practices [15,16]. The articulation between forms fixing-up micro and macro levels is made to better apprehend political and social stakes of the health system. This critical reading tackles modalities for training and allocation for human resources within the health system and the forms of distribution of care needs. This diagram is not a simple picture structuring health systems. It allows also dialectical approach between the analysis of (in)adequacy levels of the human resource management in health system and of the inequalities in the heart of these inadequacies, that is to say, their differentiated distribution in the populations and spaces.

Lastly, the reading of the diagram can and should be done with flexibility, without giving an advantage to a particular input except actors, observers, and readers' experiences. This detailed presentation of adequacy levels does not answer to a specific order. On the other hand, for each one, this article underlines initially specificities before discussing them and opening on its links with other constitutive levels of adequacy of the overall structure. This somewhat formal rigidity is meeting concern to facilitate the legibility and understanding of selected adequacies but not to reify adequacy forms and their categories of actors in the heart of the adjustments' management between offer and needs in terms of human resources for the health systems. This management in sectors of training, planning or yet of accessibility accounts for the necessarily evolutionary structure of health systems as well as included elements and links.

### Adequacy levels for human resource management in health

**Figure 1:**
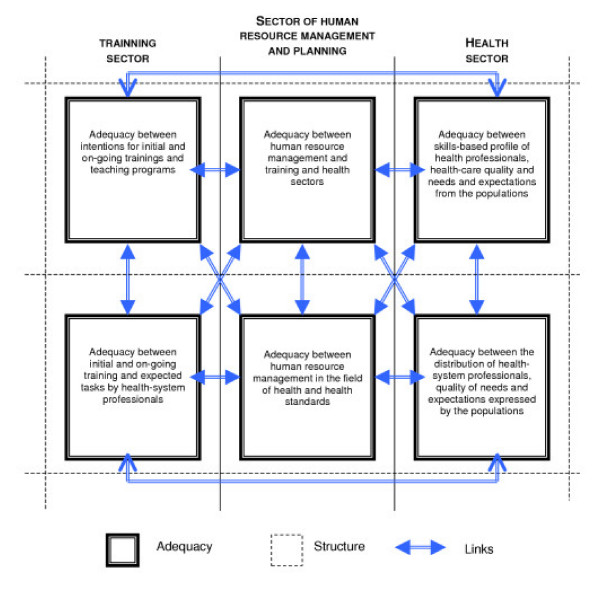
Grid of analysis for adequacy levels improving Human Resource Management in the field of Health.

#### Adequacy between the skills-based profiles of health professionals, quality for health care offers, services (health system standards), needs and expectations expressed by the populations

Within the development of health system schemes at the international level [17,18] the States and their Ministry for Health define their own health system standards according to local contexts (economical situation and structural records). These standards establish, in particular, relevant conditions of assigning activities between health care centres and the population, within the decentralized framework of health districts, and first referral hospitals located within or near the district. Without targeting a strict correspondence between population expectations and its needs, the qualitative adequacy between these two realities, established by health professionals and local epidemiological priorities, is essential to ensure an effective reference to health care structures. It necessitates accessibility being facilitated according to perception and acceptance by actors of the qualitative normative frameworks.

The standards should highlight measurements and needs expected from the population and health system professionals inclusively. Experts should be confronted with problems of priority health defined at the national level for populations in a given territory and time. These priorities can be defined according to practical orientations, in technical acts and expected activities of health professionals. Acts and resources can be specified according to categories of professionals, by developing skills-based profiles. The management and training for human resources are considered in the overall organization of the health system. It thus appears essential to check from experts and even from the population for the adjustment of health standards and skills-based profiles towards the reality of situations experienced and perceived by actors, in particular the public and primary health care professionals.

To question the adequacy of standards within the health environment with the reality of health problems encountered and perceived by the population, requires to meet actors during thorough qualitative surveys while making it possible to better determine their needs and the expression of their expectancies in order to integrate them in the development process of health standards. In Africa, these standards are still more often built without taking into account elements such as mental health or the role of tradi-therapeutics. The evolutions of the health standards should be adapted to problems experienced and felt by the populations (or defined through the health system as the new vertical programs). The qualitative adequacy of standards to the needs of societies is not, in fact, a static question but engages a progressive and continuous research/action. If health standards are a qualitative framework of reference, their definition and performances, open towards adequacy levels in more quantitative terms and whose implementation depends on available resources and appropriate needs.

#### Adequacy between the distribution of health system professionals, quality for health care offers, services (health system standards), needs and expectations expressed by the populations

Health standards quantitatively define modalities for human resources allocation within health districts in accordance with minimum packages and complementary activities. If situations are generally optimised by national health authorities, they will be conditioned by country capacities not only for human resources, but also for their management.

It is necessary to question not only the qualitative adequacy of health standards towards the needs expressed by populations, but also their quantitative adequacy according to available human resources in the sector. If standards are not adjusted to this reality, then it would be preferable to refer to more specific health references rather separate from real conditions of health care practice.

To favour these adequacies, complementary mechanisms should be considered like the development of professionals' skills-based profiles of the management. These mechanisms can in return validate or revise health standards.

#### Human resource management adequacy in health system versus health standards

Concretely, the question of the adequacy of qualitative and quantitative health standards with human resource management operates the passage of their definition according to an ideal situation with that of their definition as reflection of the national, provincial, regional, health district capacity according to the considered degree of decentralization.

This approach, first testing of field-based realities, requires a detailed inventory of human resources in health system. This thorough knowledge should be a priority in central Africa. Failing this it is difficult to set planning for human resource management. In certain situations, the setting-up of a Medical Association and Nurses Council will improve this knowledge and with it the adequacy required, since one of the first work of these organizations is its manpower census.

It is only from these records that progress can be made in terms of objectives, operations and health structures performances, in particular in terms of articulations between educational and health fields regarding the staff management (diplomas, ethical issues).

#### Adequacy between human resource management in the field of education/teaching/training and the needs expressed by health and education sectors

The planning of health sectors often considers exclusively its work in terms of management concerning its own human resources without any perspectives of coordination regarding human resource management in health and education system. This coordination however appears necessary insofar as the health and education sectors provide their contribution to the improvement of health care and services quality.

The adequacy between staff managements concerned with health and education remains essential indeed. It relates to learning modalities to reach the qualitative objectives for training and health care recommended by health standards. Thus a learning/teaching ratio in liaison with the teaching mechanism of the initial education should be referred to the existing resources in the field of education in order to adapt teaching structures to the real possibilities of the country and to consider the close links between trainings and health care quality regarding the staff management.

#### Initial and on-going trainings adequacy versus realities of the expected tasks and implemented by different categories of health system professionals within the health system organization

For a category of health professionals, the adequacy raises the issue of similarity of technical and professional education of the associated or technical level of education. This priority by category of actions is justified by the strong implication of these nursing personnel within the primary health care where they are in charge in certain contexts of more than 85% of the health care offer and services. Vis-à-vis to their important role in care practices, methodology in educational engineering defines a rather high and full profile of skills, that is to say, at the same time general practitioning and technical. The training adequacy to the implemented tasks for a category of health professionals, underlines the importance of health district as a body belonging to a coherent organization, as a first level of achievement within the field-based Human Resource Management, their inter-articulations, in particular according to categories of training and/or vocational identities which constitute the diversity of a health system.

The relocation of these adequacies by and between professional categories in the global vision of changes for the improvement of care and the effectiveness of health systems prevents professional conflicts of identities while supporting the inter-sectorial approaches. The on-the-job training should be finally adjusted with the realities on the field. The difficulty is then the planning of trainings, which has to be coherent between different professionals, and be integrated in the action plans of the health districts. The on-going training is part of vertical management programs with basic skills required by different health system professionals. The difference between the needs for care and professionals' skills are probably due to the belief by the teachers of a spontaneous transfer of knowledge in situations.

#### Adequacy of initial and on-going trainings' intentions versus teaching programs in health sciences for the trainers (teachers/supervisors/health professionals/Heads of establishments...)

In practice, one notes a lack of on-going training for teachers in many vertical programs. An aggravation of the dichotomy, established from the beginning by the inadequacy of teaching programs in health sciences, is proven by on-the-job trainings. It can be a question as well of updating vertical programs as actualising them on the concepts of primary health care and organization of health system.

Improving programs assumes reinforcing links between training objectives for future health-system professionals and teaching mechanisms in classroom as well as in the field of operation. Assessments and training plans for various levels of trainers are preconditions to any modification of program.

Beyond individual investments in training future health-system professionals, the systemic approach of actors opens a way towards an overall perspective of places and levels for trainings: from the hospital to the community, through health centres and the numerous administrative bodies. The sustainability of a teaching innovation necessitates some knowledge of adequacies by the team of trainers who ensure the follow-up and extension for changes adapted to the contexts of their achievement.

## Discussion

An overall vision remains essential for a qualitative improvement of the health care and services' system. A participative and representative process of the diversity sectors at various levels of organization plays a role in better adequacies considered not only in their specificity but also in comparing the ones to the others between and within micro, meso and macro levels [19]. Among those actors at three organizational levels (Health – Education/Planning – Human Resource Management), the question is the supporting of a common vision for change. This common horizon should come with change and even precede it. It is necessary that decision-makers and health experts from various adequacies share a same objective for change beyond specific modalities of its establishment depending on categories of actors and their level of intervention within the health system. For the decision-makers, it is a question of validating health standards for planning and programs in the contexts and conditions of their implementation. For the experts, it is a question of implementing more these planning and programs standards as effectively as possible. These are carried out and observed during trainings, practices and recourse to health care. The experts take part in the development of health-system standards while the decision-makers integrate practical field-based knowledge in the modalities for change.

Beyond field-based surveys included in its construction, the model is carrying a vision for change of the health system in all its dimensions and likely to be adopted by all the actors who share the standards and participate to its dynamic. Admittedly, in the programs presented, the attention focuses on actors at the peripheral level, nurses and health-system professionals, who are in daily contact with the health care demand and offer. However, these actors are also apprehended by their registration, their articulations with the selections, and inclusive standards of the health care system.

It is less a question of identifying a presumably adequate level of improvement relating to health systems similar to what seems to be limited to health district, than to release an overall and contextualized perspective of requirements and stakes regarding this improvement. By not taking into account the human resource management from its economist or technicist dimensions, the approach by its adequacy levels within the health systems does not take for an operational level, or even of privileged observation. The adequacies are included within the interactions between the different spatio-temporal levels of health-system organization in sub-Saharan Africa.

These spacio-temporal articulations, especially between daily and localized health practices and higher levels of development of health-system standards as educational for the organization of health care systems and their personnel, were already explored in Africa in particular by health-system anthropologists and sociologists [16]. Along with these works and the operational dimension given to the research, the identification and improvement of adequacy levels and modalities for human resource management adopt a framework of analysis dimension or level of operation. Being of multi inter-sectorial levels, this flexible model is distinguished from approaches focused on "robustness" of health districts to propose dynamic research/actions. It is no more a question of proceeding by fragmentation, neither by (de)limiting fields of operation and thinking field level in health districts, nor by artificial distinction of fields for human resource management (educational, technical, economic, sociological, political dimensions...).

The framework of analysis is not a conceptual abstract tool or "self-sufficient" recommending to improve health care structures. The health district remains an essential framework because it is a localized territory and a tool of implementation for the health system. It is in the heart of relations between actors, their aspirations and health organization, especially training for staff. The stakes for a greater effectiveness of health systems in Africa are located inside these interrelationships and their adjustments. The setting of adequacy is not conformed to modalities of implementation based on transfer of models and health standards developed international and national levels. For the development of health systems, the adequacy requires the advisory and participative approaches of all related actors at various space and time scales. The levels of decision, operation, development or evaluation are neither opposite nor juxtaposed between them. The level of health districts is, however, reinforced but not rigidified or isolated. It is approached not like a health system asset in sub-Saharan Africa but as a production space for health practices in permanent construction.

## Conclusion

Health like education is frequently presented as "everybody's business" in the 21st century societies. However, these two fields are often treated separately. The sub-Saharan African societies are representative of this dichotomy while, more than elsewhere, the poverty of the greatest population results in a poor adequacy and effectiveness between supply of care and its demand expressed by the populations. Improving primary heath care by the improvement of human resource management focuses, less on questions of means, decreased since the 1980 decade within the framework of the States of sub-Saharan Africa, than on links between their modalities of management, training and planning [20]. For years it was thought that human resources were only one aspect of dysfunctions of health districts, it is now evident that their modes of organization, in their technical, economical, educational, political, and sociological dimensions are the levers of real changes [21]. The merit for the framework of analysis proposed is to make available to various actors an overall perspective of the system to facilitate the emergence of a common vision for a more effective, socially and politically more coherent model. It recalls that behaviours of the actors, at the origin of the majority of health system dysfunctions are determined by factors amongst others educational, which are not only environmental or institutional. To act on human resources is therefore, to act on health system as a whole and thus to consider a real and sustainable impact.

## Competing interests

The author(s) declare that they have no competing interests.
